# An Overview on Carbon Quantum Dots Optical and Chemical Features

**DOI:** 10.3390/molecules28062772

**Published:** 2023-03-19

**Authors:** Marco Giuseppe Giordano, Giulia Seganti, Mattia Bartoli, Alberto Tagliaferro

**Affiliations:** 1 Department of Applied Science and Technology, Politecnico di Torino, Corso Duca degli Abruzzi 24, 10129 Turin, Italy; 2 Center for Sustainable Future Technologies (CSFT), Istituto Italiano di Tecnologia (IIT), Via Livorno 60, 10144 Turin, Italy; 3 Consorzio Interuniversitario Nazionale per la Scienza e Tecnologia dei Materiali (INSTM), Via G. Giusti 9, 50121 Florence, Italy; 4 Faculty of Science, Ontario Tech University, 2000 Simcoe Street North, Oshawa, ON L1G 0C5 T, Canada

**Keywords:** carbon quantum dots, nanomaterials, optical properties, graphene quantum dots, carbon nitride dots, polymeric carbon dots

## Abstract

Carbon quantum dots are the materials of a new era with astonishing properties such as high photoluminescence, chemical tuneability and high biocompatibility. Since their discovery, carbon quantum dots have been described as nanometric high-fluorescent carbon nanoparticles, but this definition has become weaker year after year. Nowadays, the classification and the physical explanation of carbon quantum dots optical properties and their chemical structure remain matter of debate. In this review, we provide a clear discussion on these points, providing a starting point for the rationalization of their classification and a comprehensive view on the optical and chemical features of carbon quantum dots.

## 1. Introduction

Carbon materials have undergone several revolutions during the last century [1]. Several exciting discoveries have brought carbon science beyond the traditional use of graphite and carbon black. The rise of nanostructured carbon allotropes such as fullerene [2], carbon nanotubes (CNTs) [3] and graphene [4] has paved the way for a new era in material science. Curiously, fullerenes and CNTs were first observed by accident. This also happened for an entire new class of materials: carbon quantum dots (CDs). In 2004, Xu and co-workers [5] worked on the purification of oxidized CNTs using electrophoretic techniques and isolated a highly fluorescent fraction. This was the first description of CDs, even if a proper and clear identification had to wait until the work of Sun et al. [6]. Since then, CDs have gained great attention from the scientific community due to their high photoluminescence, solubility and size [7,8]. CDs have emerged as an easier to produce and handle alternative to inorganic quantum dots due to the fast and easy preparation routes [9] and several appealing features such as biocompatibility [10], solubility [11] and optical properties [12]. CDs have been tested for several kinds of advanced biological applications such as bioimaging [13,14], drug and gene delivery [15,16,17] and theranostics [18,19]. Furthermore, CDs can be used for other applications such as catalysts [20,21,22,23,24], sensing [25,26], environmental remediation [27,28] and filler for composite production [29].

The scientific community uses the term CDs to describe a quite heterogeneous set of nanoparticles with different chemical and optical features and only two common characteristics: nanometric size and high yield photoluminescence [30]. This loose definition is one of the issues related with carbon dots together while the relation between their properties and their chemical structure represents a major challenge in the field of a rational design of CDs [31]. These unfulfilled tasks hindered a proper critical approach to CD synthesis, and aimed to optimize their interactions with biological systems or to maximize their exploitation for targeting applications [32,33]. 

In this review, we discuss the more unclear items regarding the CDs’ realm facing the issue of classification, the physical origin of their photoluminescence emission and their chemical structure. We provide a solid overview of the current state of the literature, critically enlightening the remaining unsolved issues and explanations provided by the most outstanding research in the field. 

## 2. CDs: A Classification Issue

CDs is the accepted name for a heterogeneous class of high-fluorescence nanomaterials that comprise very different species. Accordingly, the classification of CDs is matter of complexity and it has not yet been fully accepted in all of its aspects. A first CD classification is merely production-based and encompasses two groups: top–down and bottom–up procedures [34]. The top–down approach converts bulky carbon precursors into a mixture of compounds in which CDs are isolated through purification techniques. The bottom–up route is closer to traditional synthesis in which small organic molecules are combined to form CDs. Both the routes require a further purification step generally performed through dialysis or other procedures such as chromatography [35,36].

**Figure 1:**
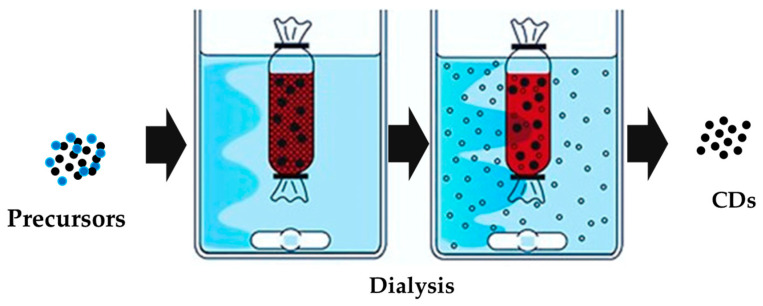
Dialysis purification of CDs for the isolation adapted from Lo Bello et al. [25] under Creative Commons Attribution License 4.0 (CC BY).

As shown in Figure 1, the dialysis process is based on the use of semi-permeable membranes that promote the formation of an osmotic pressure gradient achieving the removal of small molecules [37].

The purification of CDs through dialysis allows one to remove the small cluster by concentrating the solution outside the membrane, refining the molecular weight and size distribution of the CDs. Nevertheless, dialysis could be not sufficient for a proper CD purification and size exclusion chromatography could be necessary to fine tune the CDs’ size [35,36]. As reported by Hinterberger et al. [38], chromatography was used for fractioning CDs, concentrating the high-fluorescence species into specific fractions. Other techniques such as solid-phase [39] or cloud point [40] extraction can be used to perform the selective recovery of the fraction with specific properties of solubility and PL. Nevertheless, the choice of purification methods should be based on a solid chemical ground to properly orient the process conditions.

Therefore, the productive route-based classification can find some applications for orienting the work activity but fails to describe the CDs’ complex features. 

**Figure 2:**
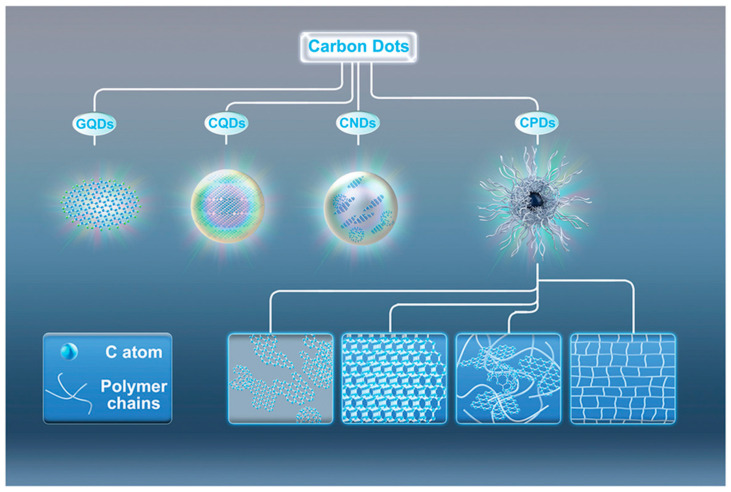
Classifications of CQDs as reported by Hia et al. [41]. Picture reprint under Creative Commons Attribution License 4.0 (CC BY).

A more comprehensive CD classification is based on their structural features, as reported in Figure 2, considering the chemical structure of core and shell regions. 

Considering the core and shell organization, Hia et al. regrouped CQDs into four classes: (i) graphene quantum dots (GQDs); (ii) carbon quantum dots (CQDs); (iii) carbon nanodots (CNDs); and (iv) carbon polymeric dots (CPDs). GQDs represent the most consistent group containing graphene oxide layers packed together [42]. GQDs are water-soluble fluorescent nanoparticles retaining the graphene lattices within the interlayer defects due to the presence of oxygen atoms [43]. The differentiation between GQDs and CNDs is less clear and tentatively based on the synthetic approach in which CQDs are produced by the carbonization of precursors, while CNDs are produced through other routes. As becomes evident, this classification is inconsistent because it is not based on particular chemical features and materials defined as both GQDs and CNDs could overlap with other CDs. As matter of fact, it is more consistent to leave behind both the GQD and CND definitions, and it is useful to introduce the carbon nitride quantum dots (CQNDs) instead [44]. CQNDs are similar to GQDs but these contain nitrogen-doped graphene units instead of oxygen-based ones, altering the core structures resembling multilayer C_3_N_3_ materials. The similarity between GQDs and CQNDs could suggest a possible broader regroup, considering all hetero atom-doped GQDs (xGQDs, where x is N, O, B, S, Si, etc.) even if the studies in the field are limited [45] compared with CD studies. 

The CPD group is the most heterogeneous one among CDs, containing species with unrelated structures but lacking a graphene-like layered structure [46]. CPDs can be produced from the thermal and chemical degradation of synthetic and natural polymers or by the condensation of organic molecules [47]. Accordingly, CPDs could be sub-regrouped in many categories based on the presence and type of heteroatoms or the precursors (polymers or small organic molecule). There is no common profile that could be used to precisely give a detailed organization of CPDs, which remains the most debated group of CDs.

The other CD classification adopted is related to their properties such as fluorescence emission wavelength [48], conjugation with biomolecules/metals frameworks/nanostructures [49,50,51] and functionalities [52,53]. The classification based on fluorescence emission is of particular interest for the selection of CD application. However, this classification could cross over the classification based on synthetic routes and structural features, creating ambiguities in the identification of a CD species. As an example, both CPDs and GQDs can be synthesized with both top–down and bottom–up approaches [41,54,55]. Furthermore, properties-based classification could regroup materials with very different structural features based on only one property such as emission wavelength [56]. The structure-based classification has several advantages compared with the other proposed schemes, but requires intensive characterization. Raman spectroscopy [57] will be used to evaluate the presence of the graphitic domain by identifying and quantifying the so-called D and G peaks originated from the defects and basal modes of graphitic domains [58,59]. Raman spectroscopy does not provide compete information, hence it must be coupled with techniques which are able to provide information about the chemical signatures to discriminate between GQDs and CNDs [60]. The most effective approach is based on the use of Raman spectroscopy together with infrared and x-ray photoluminescence spectroscopies. The combination of such techniques provides a complete overview of CDs’ composition and is useful for the definition of xGQDs.

**Figure 3:**
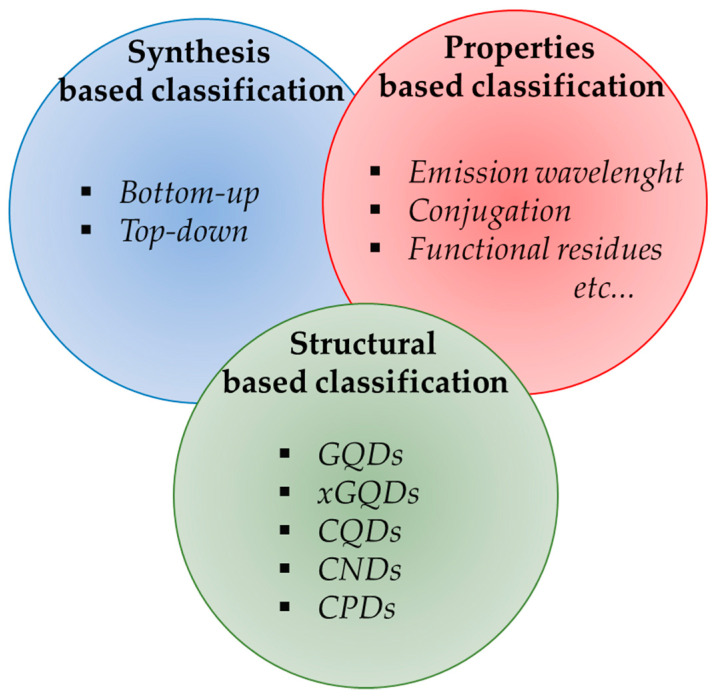
Scheme of possible classification of CDs based on synthetic approach, properties and structural features.

The brief overview of the CD classification (Figure 3) highlights the problem for the scientific community to find a common unique language for describing CDs and even the International Union of Pure and Applied Chemistry Gold Book was not able to come up with a proper definition for CDs [61]. 

Nowadays, the CD classification issue is generally ignored and scientists prefer to classify CDs following their contingency, contributing to the ambiguity of CD classification. This approach has led to a situation in which the CD label applies to every single carbon-based nanoparticle with a high fluorescence emission. This issue is detrimental to the clarity of communications related to CDs and requires a decisive effort to be solved. We believe that the structure-based classification could be a more appropriate approach to unambiguously classify CDs even if the CPDs classification remains debatable.

## 3. CDs’ Optical and Chemical Characteristics 

### 3.1. Optical Properties

The most interesting and appealing characteristic of CDs is the great intensity of their fluorescence emission [62] that originates from quantum confinement (QC) occurring when the exciton’s Bohr radius is bigger than the average size of CDs [63]. The nanometer size of carbon dots does not allow the formation of crystal-like conduction, and the valence bands and the electronic level are discrete although somewhat broadened. The HOMO–LUMO gap increases when the CD size decreases, leading to the emission of photons in the UV region with an improvement in the quantum yield (QY) [64]. The fluorescence emission for CDs with π-domains of larger size (i.e., GQDs, CQNDs) is mainly due to conjugated π-electrons of the aromatic carbon domains [65]. Larger π-domains reduced the HOMO–LUMO gap and red shifted the fluorescence emission peak [62]. The same consideration holds for all xGQDs, although some adjustments are needed because of the presence of different heteroatoms. 

**Figure 4:**
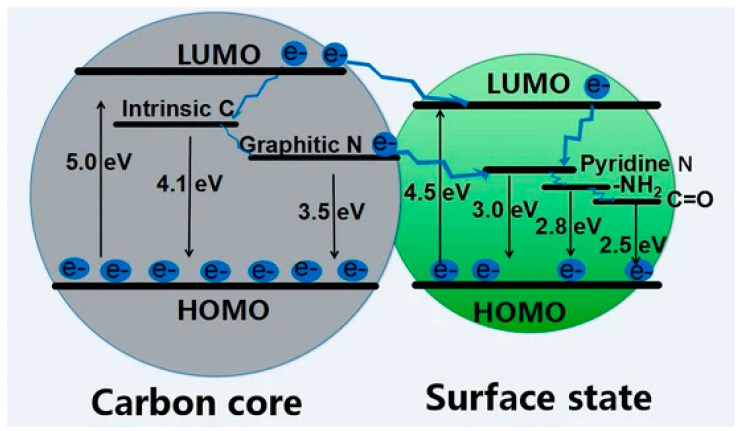
Scheme of the fluorescence emission of CQNDs based on different core/shell size ratios and surface residues. Reprinted with permission from Yu et al. [66] under CC BY 4.0.

Considering CQNDs, Yu et al. [66] investigated another interesting effect on fluorescence coming from the core/shell size ratio and surface residues suggesting the fluorescence emission mechanism reported in Figure 4.

CDs’ core-related fluorescence is not the only emission mechanism as surface states also play a role. The great complexity of surface defects and related states is reflected by the various simultaneously active mechanisms such as excitation-dependent luminescence and pleochroism. As reported by Yan et al. [62], shell surface defects are capture centers for excitons and promote radiative relaxation from excited states to the ground state, leading to multicolor emissions, the red-shift being determined by the oxidation degree [67]. Du et al. [68] suggested that shell functional groups bonded to the edge of honeycomb carbon fragments (i.e., hydroxyl, carbonyl, carboxylic residues and their heteroatom derivative) are at the origin of the visible fluorescence in xGCDs. The authors hypothesized that amides play a major role in the blue emission while carboxylic derivatives induce a redshifted emission.

CPDs fluorescence is closely related to the shell surface fluorescence of xGQDs due to the absence of a proper graphitic core. Accordingly, the aromatic clusters are dispersed and connected to each other through sp^3^ orbitals and are deemed the source for fluorescence emission [69]. As a matter of fact, the energy levels and electron cloud distribution of π and π* states in the aromatic domains could be influenced by their interaction with σ and σ* states of sp^3^ carbon surrounding [46]. Nevertheless, the great variability of CPDs requires a more detailed discussion that also considers structural and chemical features to provide a comprehensive picture of fluorescence emission. CPD fluorescence could arise from fluorophore residues retained or formed during the synthesis [70] of a heteroatoms rich regions. As reported by Qu et al. [71], nitrogen-enriched CPDs showed an increment of QY while other elements such as sulphur [72], boron [73] or fluoride [74] could increase the HOMO–LUMO gap, enhance the charge transfer or stabilize the fluorescence emission in a wide pH range, respectively. Nonetheless, CPDs could be fluorescent even without the presence of aromatic domains due to the crosslink enhanced emission effect. This phenomenon was firstly described by Tao et al. [75] by comparing the fluorescence emission of poly(ethylenimine) with CPDs-derived materials produced by the degradation and crosslinking of the polymer. The authors reported a magnification of fluorescence emission and they suggested that this was due to the formation of a rigid structure able to forbid the intramolecular rotations promoting the fluorescence emission. This was supported by further studies, indicating that the mobility reduction of the covalent bond due to steric hindrance or supramolecular interactions could further improve the fluorescence of CPDs [76,77]. 

Several mechanisms of CD chemiluminescence constitute the pillars of numerous different applications. CD fluorescence could be used for two main different applications, one based on its quenching and one based on its magnification. 

CD fluorescence quenching represents a profitable analytical tool for the detection of metals [78,79] and organic species [80]. The mechanisms of fluorescence quenching are multiple and are related to the interactions between CDs and the quencher molecules. The quenching mechanisms are (i) dynamic quenching [81], (ii) static quenching [82], (iii) photoinduced electron transfer [83], (iv) Förster resonance energy transfer [84], (v) Dexter energy transfer [85], (vi) inner filter effect [86] and surface energy transfer [87].

The dynamic fluorescence quenching is due to a diffusion quencher while CDs are in their excited state. The fluorescence dynamic quenching of CDs is temperature-sensitive and can be used for the detection of both inorganics and organic compounds [88]. Gonçalves et al. [89] attributed the CDs’ quenching observed in the presence of Hg(II) to the dynamic quenching effect due to the intensity of the phenomena without supporting it with more detailed investigations. A similar consideration was reported by Wang et al. [90], suggesting that the simultaneous dynamic and static fluorescence quenching. Contrary to dynamic one, static fluorescence quenching occurs through the formation of a ground state complex between the quencher and CDs [91]. The static quenching of CD fluorescence is tentatively exploited for the detection of inorganics that are forming stable complex species with CDs. Xu et al. [92] reported static quenching for poly(ethylene glycol)(PEG)-coated CPDs in the presence of Pt(IV) and Au(III). The metal–shell interactions promoted a great quenching of native fluorescence emission intensity without altering the fluorescence decay pathways. Authors suggested that it was due to static quenching involving the absence of electron–hole radiative recombination. Alternatively, to monitoring fluoresce quenching, Gong et al. [93] prepared quenched CPDs by creating stable complexes with Fe(III). The quenched CPDs were used to detect ascorbic acid due the formation of iron ascorbate, removing the quencher from CPDs with a corresponding enhancement of fluorescence emission. Wang et al. [94] proved the ability of CQNDs to form stable complexes on the shell with several metal ions through complex formation with nitrogen-based residues. The interactions between metals and CDs could also be destructive as reported by Song et al. [95]. The authors showed that, in presence of oxygenated water, Fe(II) could promote a Fenton reaction with the chemical oxidation of CDs.

Photoinduced electron transfer quenching occurs on distances greater than 10 nm between the quencher and CDs [96], while the Förster resonance energy transfer quenching takes place for distances lower than 10 nm and involves dipole–dipole interactions [97]. These three phenomena were investigated as possible new routes to convert photons into electrons for the production of molecular devices [98,99]. Nonetheless, Förster resonance energy transfer has found an interesting application in the development of a dual species probe composed by CDs and metals clusters [100]. This approach allowed to quench CD fluorescence and then reactivate it when metal clusters are removed by the presence of analytes. 

The distance between the CDs and the fluorescence-quenching mechanism is also affected by the condition of the CD system. Yoo and co-workers [101] investigated the effect of dilution on GQDs with a controlled carbonization degree. As stated above, the fluorescence emission of GQDs is mainly due to π-conjugated domain of the core but the surface states play a relevant role in promoting the quenching aggregated state. This dual role of the π-conjugated domain in CDs offers a challenge to the prediction of photoluminescence (PL) characteristics in the solid-state. By tuning the core–shell ratio and the aromatic domains size, the authors proved that the CDs with a higher core–shell ratio of π-conjugated domain are dominated by a core emission in the concentrated solution with the total suppression of surface emission. Dexter energy transfer quenching occurs on a similar scale but it is based on orbital overlap [102]. The inner filter effect was originally considered as an error in fluorescence measurements, but it was not. It is a particular phenomenon due to the overlap between the excitation or emission spectrum of CDs and the absorption spectrum of quencher [103]. Surface energy transfer quenching takes place more in quantum dots that in CDs and it is due to interactions between surface plasmons and the orbital system of a fluorophore [104]. Nonetheless, it can also occur between CDs and small metal clusters [105]. 

An interesting phenomenon related to CD fluorescence is the excitation-dependent emission and its origin is debated in several studies [106,107]. A relevant study was conducted by Krishnaiah et al. [108] using a top–down approach to hydrothermally convert blue grass into CPDs. Authors reported the remarkable redshift of a fluorescence emission peak from 370 to 470 nm using a wavelength ranging from 280 up to 400 nm. The authors hypothesized that the red shift occurred due to n→π* transitions between non-bonding molecular orbitals of carbonyl and carboxylic groups on the CPDs shell. This was in good agreement with the modelling of PCDs’ structures reported by Mintz et al. [109] and with the nitrogen doping detected. The performances of PCDs were reasonable, with a quantum yield of 7% and the ability of detecting of Mn(II) and Fe(III). Surprisingly, the authors detected a loss of fluorescence quenching in the presence of both Pb(II) and Cd(II) suggesting the poor complexability of these cations by the functionality residues of the shell. The excitation-dependent emission is rather challenging due to the violation of the Kasha–Vavilov rule [110] and a first attempt to provide a theoretical explanation was provided by Khan et al. [111]. The authors investigated the fluorescence of CQNDs by a nanosecond time resolving spectroscopy reporting a significant energy redistribution and a relaxation among the emitting states. The authors proved that the inhomogeneous broadening and red shift of the emission peak were due to a system of energy sub-states and not caused by a quantum confinement of a different-sized CQNDs [112] and only weakly related to the oxidation of shell edges. This last point is still debated and the other authors suggested that the red shift was mainly related to the shell oxidation degree [113]. Experimental results are inconclusive and we believe that both interpretations are possible even if they reached opposite conclusions. This is related to the variability in CQNDs structures and unique features that make it impossible to provide a unique explanation for the emission-dependent red shift and require a two-limit scenario as mentioned above, which is able to describe all the CQND materials produced using different routes. 

CDs could also show another photoluminescence mechanism: phosphorescence [7]. Phosphorescence originates from the intersystem crossing from the lowest excited singlet state level to the triplet state and from radiative decay from the lowest excited triplet state to the ground state one. Particularly, PCDs are quite effective to exploit phosphorescence due to the crosslinking between aromatic domains and supramolecular interactions, RTP can be easily achieved through appropriate design without additional matrices, due to covalently cross-linked frameworks, polymer chains and supramolecular interactions [114]. Tao et al. [115] systematically studied the PCD phosphorescence, showing that the crosslinked matrix suppressed the nonradiative transitions due the covalent bonding of the neighboring emissive centers. Accordingly, the energy level distributions reduced the energy gap compared with the precursors promoting the formation of triplet states. Furthermore, the complex network of hydrogen bonds contributed to the system rigidity, decreasing the nonradiative relaxation. Phosphorescence was also observed in xGQDs: Song et al. [116] exploited the UV phosphorescence of CQNDs by reducing the graphitic domains or reducing the mobility of CD domains by adding sodium isocyanate crystals. The authors proved that the electron transition from the p_x_ to the sp^2^ orbital of the nitrogen generated an orbital angular momentum able to populate the triplet state. At the same time, the encapsulation of CQNDs reduced the energy dissipation triplet excitons reaching a phosphorescence lifetime of up to 16 ms. Similar results could be achieved by entrapping CQNDs into the silica [117] or polymeric matrix [118]. Knoblauch et al. [119] followed a different route to achieve phosphorescence CDs based on tailoring with bromine. Bromine-doped CDs had accessible triplet states exploiting the phosphorescence in liquid media at room temperature. A key factor that must be considered for the preparation of phosphorescent CDs is the stability. As discussed in the review work of Wei et al. [120], long exposure to UV light induced a loss of shell residues leading to the quenching of phosphorescence. Hu et al. [121] faced this issue by tailoring the CD surface with silicon residues, prolonging the lifetime under hard UV light up to 200 h. 

Another relevant optical property of CDs is their chemiluminescence [122]. Chemiluminescence is produced during chemical reactions in which intermediate radical species decompose to form electronically excited species and deactivate themselves. CD chemiluminescence originates from the fluorophore residues that act as emitting centers, as first reported by Lin et al. [123]. CD chemiluminescence has found plenty of applications in optoelectronics and catalytic processes related to the various structures of CDs available with unique fluorophores and energy levels [124]. Interestingly, Xu et al. [125] reported the tunability of chemiluminescence response by tuning the oxidation degree of CDs. The authors proved that highly oxidized CDs showed a high fluorescence emission with chemiluminescence while the opposite was true for poorly oxidized CDs. The authors concluded that fluorescence was related to the core states for photon absorption with the shell acting as a long wavelength trap providing nonradiative recombination centers. Conversely, chemiluminescence was related to the radicals formed in the shell surface states.

### 3.2. Chemical Features

The chemical properties of CDs vary according to their structures, which themselves vary with the production conditions. 

**Figure 5:**
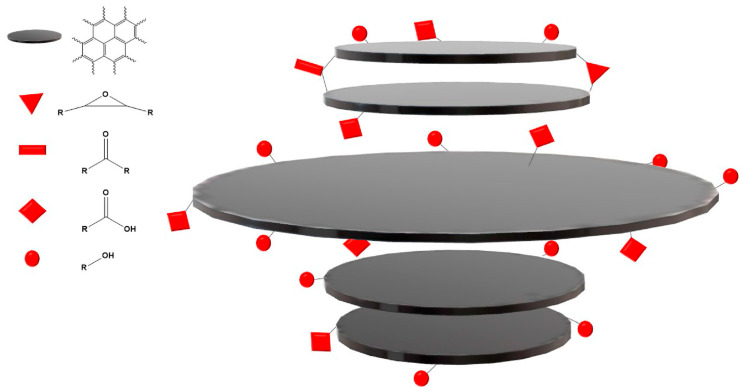
Schematic structure of GQDs. Reprinted with permission from Mintz et al. [109].

GQDs offer the simplest interpretation of chemical properties based on the Lerf–Klinowsky model for graphite oxide [126] with the structural arrangement reported in Figure 5.

**Figure 6:**
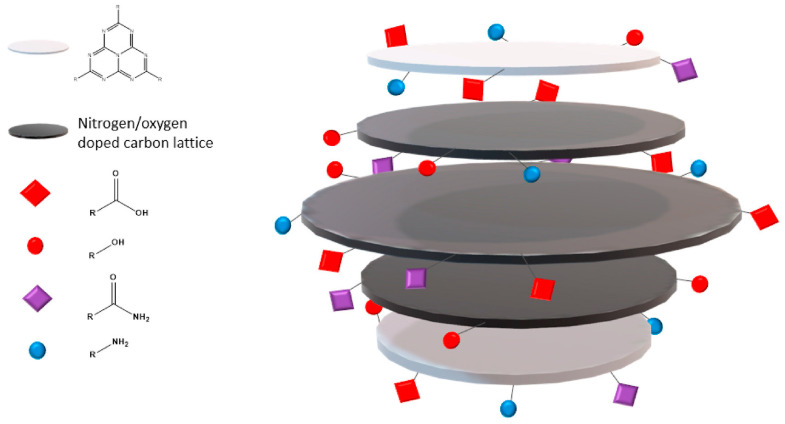
Schematic structure of CQNDs. Reprinted with permission from Mintz et al. [109].

GQDs are generally composed of a few layers of oxygenated graphene-like structure in which highly oxidized functions such as carbonyls and carboxylic ones are concentrated at the edges. GQD layers could be classified into four types, named Types 1–4, based on their morphology (triangular, rectangular, hexagonal, dendrimeric) [127] based upon which the HOMO–LUMO gap is affected [128]. GQDs are generally soluble [129] or at least dispersible in water-forming colloidal suspension [130] and can be functionalized by simply adding heteroatoms. The heteroatoms could be added as doping agents after the synthesis [131] or could be integrated into the graphene-like structure during the production of GQDs as in the case of CQNDs [132]. CQNDs are of particular interest due to the massive incorporation of nitrogen atoms into graphene-like layers, as depicted in Figure 6.

**Figure 7:**
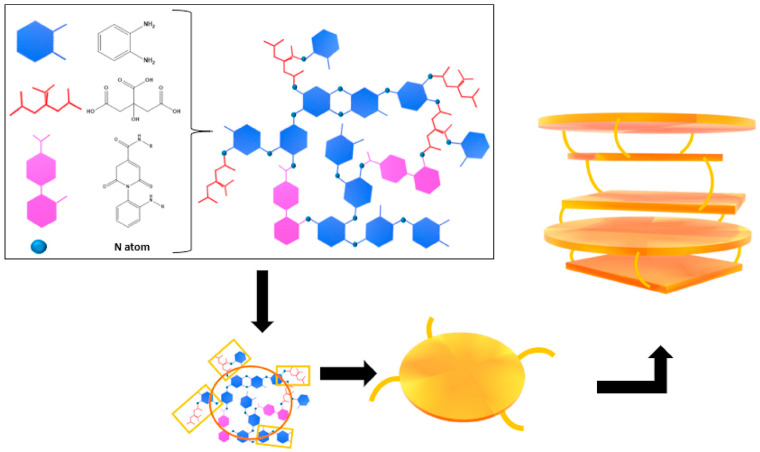
Schematic structure of PCDs produced using 1,2-diaminobenze and citric acid. Reprinted with permission from Mintz et al. [109].

As reported by Liyanage et al. [133], the structure of CQNDs is more complex than if simply originated by the replacement of oxygen atoms in GQDs with nitrogen ones. The use of highly reactive precursors such as urea promoted the formation of heptazine layers on the CD ends, as proved by the solid-state nuclear magnetic resonance investigation that sensibly changed the CD arrangement. Mintz al. [109] reported a detailed mechanism for the early stage of CQND production enlightening the key role of cycloadditions condensation for the production of nitrogen-rich aromatic domains. Furthermore, Kirbas et al. [134] showed that the variation between precursors promotes no significant morphology change but a modification in edge residues. The functionalized edges of CQNDs are the key feature for the interaction with watery media as reported by Wiśniewski [135]. The author evaluated the interaction between water and CQNDs showing the occurrence of proteolysis with the local formation of [OH(H_2_O)_n_]^−^ clusters. This phenomenon induced the formation of a nonconductive Eigen-like CQNDs–water complexes in both Stern and diffusive layers. The CPD structures are far more complex than those of other CDs and each material requires a standalone discussion for describing its unique chemical features. We can split CPDs into two main groups, one composed of CDs produced by polymers degradation and the other encompassing PCDs produced by the condensation of molecular units. The first group is characterized by the partial retention of the polymer’s original structure and their functionalities [47]. Nevertheless, these PCDs have been poorly investigated and their structural studies are generally limited to the composition of the surface residues. PCDs produced by using molecular precursors are considerably easier to characterize by modelling their reactivity. Mintz et al. [109] proposed both a formation mechanism and a structural model for PCDs produced using citric acid and 1,2-diaminobenzene, as shown in Figure 7.

The authors proved that several mechanisms acting simultaneously during the early stage of PCD production, such as the formation of amide bonds together with the partial condensation of diaminobenzene units. As clearly emerged, the authors suggested that PCDs were not a unique and well-defined species but a distribution of species with common chemical features such as their aniline units and citric acid amide derivatives. 

**Figure 8:**
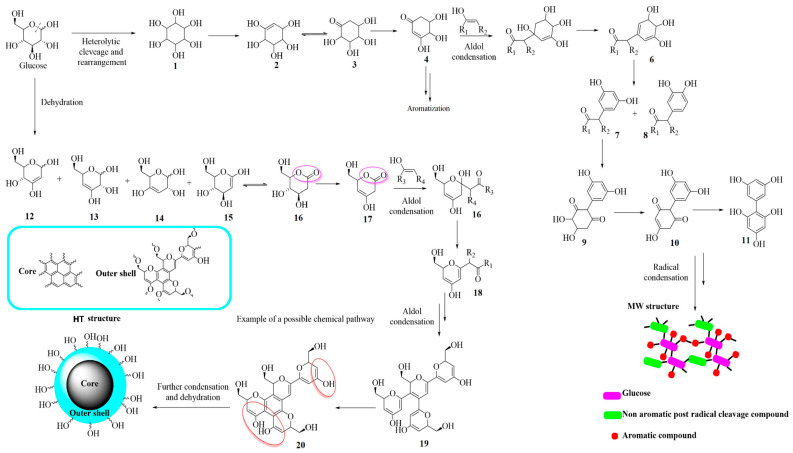
Schematic chemical pathway for the production of GQDs or PCDs using glucose as precursors and different synthetic routes. Reprinted with permission from Seven et al. [136].

PCDs’ chemistry is also deeply affected by their synthesis conditions, as reported by Seven et al. [136]. The authors investigated the formation of PCDs using glucose as a single precursor under microwave irradiation or through hydrothermal conditions showing two possible pathways, as reported in Figure 8. 

Glucose reacted very differently based on the heating procedure. As reported by Bartoli et al. [137], glucose rearranged itself through a radical mechanism forming a polyalcohol creating both (i) aromatic units through radical reactions; and (ii) condensed units through aldol condensation. These fragments were combined into a complex reticulated PCD structure. The hydrothermal synthesis promoted a totally different reactivity dominated by an aldol condensation and aromatization process induced by dehydration and rearrangements. 

**Figure 9:**
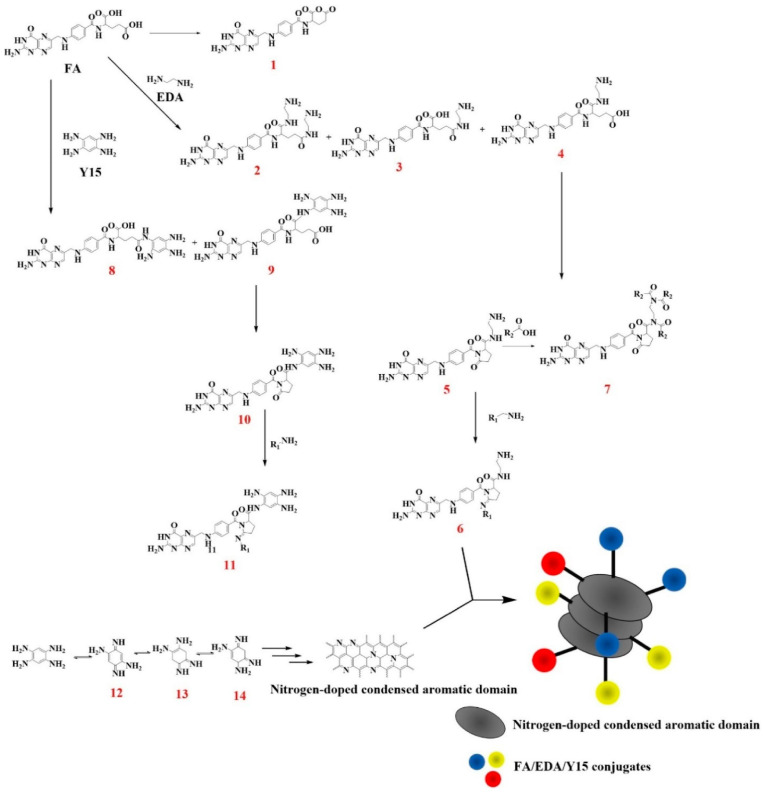
Schematic chemical pathway for the production of QCNDs tailored with folic acids. Reprinted with permission from Chen et al. [143].

Using the knowledge on CD chemical structures, it is possible to produce highly complex molecular architectures through conjugation with several species. Li et al. [138] bonded GQDs with transferrin through carbodiimide crosslinking, providing a new diagnostic tool able to cross the blood–brain barrier by endocytosis mediated by transferrin membrane receptors. The authors further functionalized the systems by tailoring the transferrin–GQDs with doxorubicin targeting and selectively inhibiting pediatric brain tumor cells in vitro [139], creating a proper theranostic tool. This system was able to selectively release doxorubicin in a slightly acid environment, pH 6.4, which is characteristic of several cancerous cell lines [140]. The carbodiimide strategy could be also used for labelling proteins [141] and bond oligonucleotides, as reported by Srivastava et al. [142]. The authors used a small nucleotide chain for coupling a red and a green emissive CDs, promoting the interaction with cellular nucleic acids. CDs could also be conjugated with specific membrane molecular receptors that are overexpressed by cancerous cell lines such as folic acid. As reported by Chen et al. [143], folic-acid-rich PCDs could be performed under microwave irradiation with a formation mechanism such as that reported in Figure 9.

Folic acid was mixed with tetraaminobenzene and ethylenediamine, producing amide derivatives, amine free chains and lactone moieties. These last two chemical species act as condensation and reticulation centers producing a shell rich in slightly modified folic acid fragments as proven by mass spectroscopy investigation. Core structures were mainly composed of condensed tetraaminobenzene units in a layer close to QCNDS providing a solid structure for the system.

Alternatively, CDs could be coupled with enzymes to monitor and guide their effects [144,145] on metal centers for high-field magnetic resonance analysis [146]. Interestingly, Zhou et al. [147] coupled GQDs with gel-like CDs in order to magnify the drug loading ability, leaving the biocompatibility and mobility in all body districts untouched. This pioneering study paved the way for multi-CD structures with tailored properties and hopefully for future CD-based oligomers and polymers. 

## 4. Conclusions

This brief review provides some useful clarifications on CDs, and a clear and concise reference point to approach the vast realms of CDs. As emerged from the literature analysis, CDs are poorly standardized and a systematic classification in the rigid library still has far to come. The research community has found a clear way to think about carbon allotropes such as fullerenes and CNTs, but is still missing a general consensus on what CDs are apart from small fluorescent particles. Here, we provided the foundation for a more systematic classification proposing the generic definition of xGQDs and PCDs as the only two classes of CDs, even if we recognize the difficulty in rationalizing PCDs groups. Nevertheless, these two categories could represent a solid base for designing new CDs with enhanced optical properties. Considering CDs’ photoluminescence, the researchers agree on the major point even though the physical mechanism of the fluorescence emission-dependent red shift should be tailored in consideration of the core–shell ratio. The advantage of a rational approach to the design of CD synthesis emerged from the number of studies we discussed in which authors focused on the proper planning and design of experiments. We believe that a similar approach towards the CDs design based on a CDs structure ground will be able to foster the development of CDs having new and tailored functionalities and abilities. 

We believe that the CDs represent an immense reservoir for new discoveries and a more rational approach to design and classification will be a mandatory guideline for any future significant development.
